# Factors influencing elderly people’s intentions to use smartphone mHealth apps: Quantitative research

**DOI:** 10.1371/journal.pone.0361137

**Published:** 2026-10-23

**Authors:** Tianyang Huang, Wenqin Cai

**Affiliations:** 1 School of Design Art, Xiamen University of Technology, Xiamen, China; 2 Department of Geriatrics, The First Affiliated Hospital of Fujian Medical University, Fujian Medical University, Fuzhou, China; 3 Fujian Hypertension Research Institute, The First Affiliated Hospital of Fujian Medical University, Fuzhou, China; 4 Department of Geriatrics, National Regional Medical Center, Binhai Campus of the First Affiliated Hospital, Fujian Medical University, Fuzhou, China; 5 Clinical Research Center for Geriatric Hypertension Disease of Fujian Province, The First Affiliated Hospital of Fujian Medical University, Fuzhou, China; BRAC Business School, BRAC University, BANGLADESH

## Abstract

Smartphone mHealth apps (mHealth apps) have played a highly positive role in medical services, which provides a new direction for users’ medical services and health management. Although smartphone mHealth apps provide many benefits for users, people know little about the factors that drive elderly users to adopt this new service platform in the context of an aging population. This study aims to learn the factors that affect elderly people’s intentions to adopt smartphone mHealth apps, and use the technology acceptance model as a theoretical basis to integrate perceived convenience, perceived quality, perceived risk and perceived trust, in an attempt to construct a research model on the intentions to use smartphone mHealth apps. The elderly users’ data were collected by questionnaire survey and analyzed quantitatively by structural equation model. By analyzing the 263 valid data collected, the results showed that users’ perceived trust (β = 0.427, p < .001), attitude (β = 0.285, p < .001), and perceived quality (β = 0.278, p < .001) of mobile medical applications positively influenced users’ intention to use the applications, while perceived risk (β = −0.173, p < .001) had a negative impact on the intention to use. Perceived trust was also directly positively influenced by perceived quality (β = 0.434, p < .001) and perceived ease of use, and perceived convenience positively influenced perceived usefulness (β = 0.358, p < .001) and attitude (β = 0.299, p < .001). These findings can help us better understand the factors that influence the adoption of smartphone mHealth apps. This study further extends the technology acceptance model and its application domains to help mHealth researchers, designers, marketers, and service providers improve mHealth services and help increase users’ intentions to adopt mHealth apps.

## Introduction

As a small, portable handheld terminal, the smartphone has brought great convenience to people’s lives, breaking the limitations of time and space and making it easier to obtain and transmit information. The development of smartphone technology has spawned the development of mHealth apps [1]. As smart technology develops and smartphones become more common, medical and healthcare mobile apps have increased dramatically [2], especially during the COVID-19 epidemic, and the advantages of smartphones for interactive communication between doctors and users have become particularly prominent [3]. The emergence of mHealth apps is. The emergence of mobile medical applications (apps) may bring an innovation revolution to clinical medicine, which is widely discussed in healthcare innovation. Mobile medical applications (apps) may revolutionize clinical medicine, creating opportunities and challenges [4]. mHealth is defined as medical and public health practices provided with mobile device support [5], including mobile healthcare applications [6]. The World Health Organization's Global Observatory for e-Health defines mHealth applications as mobile applications that collect and manage relevant medical information for users, supported by mobile devices such as mobile phones, personal digital assistants, and other wireless devices [7]. It can enhance inclusion in healthcare, expand access to health information and knowledge, and promote improved health behaviors [8]. In the first quarter of 2021, the number of mobile health apps available to iOS users worldwide has reached nearly 54,000 [9]. In Germany, 33 mobile health apps have completed certification as of June 2022 [10]. As of March 2022, there are 104,000 mobile health applications worldwide [11]. As of June 2023, China, the world’s largest developing country, had 364 million users of internet-based healthcare, an increase of 1.62 million from the past six months [12], indicating a huge market size in the future. The development of information and communication technology has continuously increased the online medical data represented by internet hospitals. According to the report, China had more than 3,000 internet hospitals that had provided internet diagnosis and treatment services for more than 25.9 million people [13], and large internet medical platforms showed good development trends. For example, Alibaba Health’s total revenue for the fiscal year of 2023 (from April 1, 2022 to March 31, 2023) is RMB 26.76 billion, an increase of 30.1% year-on-year [14]. JD Health reported revenue of RMB 13.95 billion for the first quarter of 2023, an increase of 54.2% year-on-year [15]. The use of mhealth apps is increasing rapidly, but their availability and acceptance need to be proven [16].

Gordon et al. [4] pointed out that smartphones have become an important routine accessory in healthcare clinics, and their combination with other computing technologies has gained attention for facilitating efficient interactions between doctors and patients [17]. Relevant studies [18, 19] pointed out that during the COVID-19 epidemic, COVID-19 was better prevented and controlled thanks to the convenience and other advantages brought by smartphone mHealth apps. Exactly because it has the outstanding advantage of breaking physical distance, mHealthcare received great attention during the COVID-19 epidemic [20]. During the COVID-19 pandemic, the acceptability of digital health applications may have increased due to public anxiety about potentially unsafe access to hospitals, travel restrictions, etc. [21]. Mobile health (mHealth), as one type of digital health application, has attracted significant attention in the medical care field [22]. Meanwhile, Alaiad et al. [23] also pointed out that mHealth apps provide users with online medical appointment and consultation services. They can produce effective management of patients’ chronic diseases [24] and improve users’ health. For example, Candelaria and Cacciata [25] applied personalized mobile health technology intervention to manage the risk of cardiovascular disease in elderly patients. Wang and Chen [26] demonstrated the efficacy of mHealth app-based continuous care in improving user knowledge and treatment compliance. In addition, Abbaspur-Behbahani et al. [27] pointed out that mHealth successfully improved medical and health services during the COVID-19 epidemic and is a good solution for communication between doctors and patients [28]. With unique advantages such as practicality and convenience, mHealth apps play an active role in medical services and provide a new direction for users’ medical and health management [29,30]. It offers strong scalability and can reach a wide range of users across different geographical locations [16]. Although mHealth has multiple advantages, it also presents challenges for users’ utilization and adoption of this new technology. The usage rate of users is still low [9], so its original need needs to be explored. However, considering the potential benefits of mHealth for patients, healthcare managers, and physicians, it is important to assess the factors that may motivate the intention to adopt mHealth apps [31], as using a medical app is the first step to getting it noticed [32]; however, this explains the factors affecting users’ use of medical mobile tools [31].

The behavioral decision-making of hospital patients is complex, involving not only technical considerations but also psychological perception factors of users [33]. This needs to be studied using relevant behavioral theories. The Technology Acceptance Model (TAM) is one of the most widely used theoretical models to explain the factors influencing consumers’ adoption of new technologies [34,35]. As a widely used IT acceptance model, TAM has been shown to be applicable to studies on patients’ use of mobile apps, such as a study on diabetic patients by Zhang et al. [36] and a study on users’ intentions to use mHealth apps in Indonesia by Octavius and Antonio [37]. In addition, Byrd et al. [38] used TAM as the theoretical basis, and their results showed that users’ intentions to use mHealth apps could be enhanced by improving perceived ease of use and perceived usefulness. Although TAM has been widely used and proven, and has a high explainable variance in users’ technology adoption [39], some studies [39,40] pointed out that researchers need to combine different variables to explore research on different topics and contexts. Furthermore, previous studies have focused on mHealth apps and mainly focused on medical care workers [41], nursing students [42], and pharmacy students [43]. For example, Xu et al. [44] explored the factors influencing medical students’ continuance intention to use mHealth apps, and the results showed that medical students’ perceived usefulness and attitudes had a significant impact on their continuance intentions to use mHealth apps. Galetsi et al. [45] used sociological theories to study the process of existing mobile app adoption behavior from the perspective of medical professionals, which mainly focused on social norms and trust issues. Dahlhausen and Zinner [46] investigated the obstacles to the adoption of m-health from the perspective of doctors and psychotherapists. Sidek, Ismail [47] explored the acceptability of mHealth applications among middle care workers in Malaysia from the perspective of hospital care workers.

Learning to use the internet can be challenging for people [48], and this is especially true for online medical services [49]. Although the advantages of mobile healthcare are obvious and its potential is immense, older adults often refuse to adopt it [50–52]. For example, in the Netherlands, a developed country with a high incidence of aging, half of the elderly have no intention to use mHealth apps [50,51]. Although the number of studies on the adoption of mobile medical applications has increased, significant limitations remain in research subjects, theoretical boundaries, and contextual settings. A review specifically for the elderly population indicates that existing studies mostly focus on adoption barriers, insufficient digital skills, privacy concerns, and interface adaptation issues, but there is still a lack of systematic integration and dedicated discussion on the antecedents of adoption among elderly end-users, especially those in China, particularly in terms of the adoption process [53,54]. More importantly, although existing TAM studies have been widely used in health informatics and often enhance explanatory power by incorporating contextual variables, there is still no “optimal unified version” applicable to health services, especially in the context of mHealth for the elderly, indicating that the adoption mechanisms in different populations and different service scenarios still need to be further refined and analyzed [49,55]. From the perspective of research subjects, existing mHealth studies have long focused more on general patients, healthcare workers, students, or continuous use in chronic disease management rather than the first-time or overall intention of use among the elderly; even in elderly studies, a considerable portion focuses on continuous use [49,54] of assistive technologies or broader digital health, rather than the specific carrier of smartphone mobile medical applications [56–58]. Therefore, the key difference between this study and existing work lies in: targeting Chinese elderly smartphone users, focusing on the more specific behavioral outcome of “intention to use mobile medical applications on smartphones,” and while retaining the simple core structure of TAM, simultaneously incorporating variables such as perceived convenience, perceived quality, perceived risk, and perceived trust that are closer to the attributes of medical platforms, in order to identify the key psychological mechanisms of elderly users in the pre-adoption stage.

It is worth emphasizing that the placement of this study in the Chinese context is not solely due to the fact that the sample comes from China, but rather because the willingness of Chinese elderly people to use smartphone mobile medical applications is embedded within a family structure, digital infrastructure, and healthcare environment that is different from those in Western countries [59,60]. Lupianez-Villanueva and Folkvord [31] discussed the factors that affect the adoption of mobile medical applications, but this study focused on Western developed countries (Spain, Germany, and the Netherlands). Existing research has already indicated that the adoption of health technologies by the elderly varies significantly across different regions. The geographical area alters the strength of the relationship between perceived ease of use and behavioral intention, so the adoption mechanism needs to be explained within the specific social and cultural context rather than being simply generalized [61]. The uniqueness of China is first manifested in the simultaneous advancement of aging scale and digital transformation. China has a huge base of elderly population, and the expansion of the Internet and smart healthcare is rapid, but the overall internet usage rate among the elderly is still significantly lower than the overall level, which makes the adoption of digital health have both market potential and digital exclusion characteristics [59]. As the world’s largest developing country, China has a large elderly population. According to the 52nd Statistical Report on China’s Internet Development [12], as of June 2023, the number of non-netizens in China was 333 million. By age, the elderly aged 60 and above were the main group of non-netizens, and the proportion of Chinese non-netizens aged 60 and above accounted for 41.9% of all non-netizens. These non-netizens are unable to access the internet, so they cannot fully enjoy the convenience brought by intelligent services. Therefore, given the large potential market size for smartphone mobile medical applications in the elderly population in China, a developing country with an increasingly serious aging problem, what factors may affect elderly people’s intentions to use smartphone mHealth apps at the time when smartphone mHealth services emerge? This is a question that needs to be explored. It can be said that studying the intention of elderly people to use mobile medical applications in China is not only significant for explaining the acceptance behavior of a new technology, but also for addressing the real issues of digital health equity and healthy aging in the context of an aging population [49,62]. This research not only helps to explain the formation mechanism of the elderly's intention to use mobile medical applications, but also can provide more locally targeted theoretical basis and practical references for the design of age-friendly interfaces, family collaborative support, community digital training, and the optimization of smart health policies in the Chinese context [62,63].

In conclusion, with the popularity of smartphones, mHealth applications have proven to be a low-cost and acceptable health management tool [64]. It is a widely discussed technology in healthcare innovation and has the potential to bring revolutionary opportunities and challenges to clinical research [45]. Most existing studies focus on the demographic characteristics of mHealth [65], external environment [66], non-elderly groups [44,45], user factors [67], etc. Little is known about the elderly population’s use of smartphone mHealth apps. Sykes et al. [68] pointed out that we should pay attention to the user psychology that promotes the use of technology, and only in this way can we promote the success of technology systems. Current research on the influencing mechanism of the elderly people’s intentions to use mHealth apps is still in its infancy [2]. Therefore, the purpose of this study is to explore the factors influencing the elderly users’ intentions to use mHealth apps. The research is based on the technology acceptance model and constructs a model for the intention of elderly people to use mobile medical applications on smartphones. It then analyzes the relationships between perceived convenience, perceived quality, perceived trust, perceived risk, perceived usefulness, perceived ease of use, attitude, and intention through a structural equation model. In other words, this paper focuses on how the intention of elderly users to use such applications forms after they have actually come into contact with them, rather than the initial adoption process from non-users. This study can provide some positive references for the elderly-friendly development and design of mHealth apps and mobile health, as well as healthy aging.

## Research theory and hypothesis development

### Research theory

The Technology Acceptance Model is a theory proposed in response to the rapid development of information technology to predict users’ acceptance of information technology and to promote the innovative development of science and technology. It is an excellent user acceptance model [69]. The Technology Acceptance Model proposed by Davis et al. [35] is one of the most basic models for explaining users’ adoption of a technology or service [70]. The model proposes two psychological factors -- perceived usefulness and perceived ease of use, which can predict users’ attitudes towards and intentions to use technology, and points out that users’ perceived usefulness and ease of use of a technology product or service positively affects users’ attitudes to use, and users’ attitudes to use of a technology have a positive impact on users’ intentions to use the technology. TAM focuses on and predicts users’ intentions to use technology, and it is widely used in validation studies [71,72]. Since its inception, TAM has received continuous attention, and different extensions have been proposed depending on the context [73], indicating the model's potential for evolution. As Benbasat and Barki [74] reported, TAM is one of the most influential classical theories in information systems. The TAM model has become one of the most widely used theoretical models in technology evaluation. Its predictive effect on the adoption intention of a technology system or service is undiminished [75]. Therefore, it is widely used in various fields of research. According to de Graaf and Ben Allouch [76], the widespread popularity of TAM can be attributed to at least three reasons, namely, IT is a concise and IT-specific model, the model has a solid theoretical foundation and a well-researched, easy-to-use scale, and it has a good overall explanatory power. It has accumulated strong empirical research validation support. As smartphone mHealth apps are a new service technology, Jin and Yuan [9] revealed the utility of perceived usefulness and ease of use in the use of mobile health applications. Hence, this study adopts TAM as the theoretical basis.

Although the Unified Theory of Acceptance and Use of Technology (UTAUT) [77]/UTAUT2 [78] integrates multiple competing theories and has high explanatory power, its applicability in the elderly population and in medical contexts remains controversial. Liu et al. (2023) found that over 80% of the core factors in the extended UTAUT2 model have no significant relationship with older people's intentions for mHealth use, and ultimately only trust and habit were significantly related, indicating that the original structure of UTAUT2 is not only redundant but also lacks explanatory power in the elderly medical context [79]. In contrast, the dual paths of perceived usefulness and perceived ease of use in TAM have been repeatedly verified as the most robust core mechanisms in elderly health technology adoption [61,80]. Choosing TAM as the basic framework is not due to simplicity considerations, but because its core paths provide a stable, scalable structural anchor that enables external constructs to form a coherent causal chain through “attitude” as a mediator [80,81]. Based on this, this study introduces perceived convenience, perceived quality, perceived risk, and perceived trust, not a translation of UTAUT/UTAUT2 elements, but an integrated logic centered around “features of medical service platforms”: perceived convenience and perceived quality serve as external antecedents, entering the TAM core path through enhanced perceived usefulness and perceived trust respectively; perceived risk and perceived trust act as the belief layer, directly influencing the use attitude and intention [60,79,82]. This design differs from the flat structure of UTAUT/UTAUT2, where each construct acts directly on behavioral intention, by embedding the trust-risk mechanism specific to the medical context into the causal chain of TAM, thereby forming a theoretically expanded framework with contextual explanatory power [79].

Although TAM is robust in explaining health technology adoption, systematic reviews have shown that its predictive power usually requires further enhancement through contextualized extended constructs, especially in scenarios such as mobile health applications that combine technical, service, and highly sensitive health information attributes [49,55]. Recent evidence further indicates that in mHealth adoption studies, digital literacy, trust, and application factors are the most stable extended directions. At the same time, risk-related constructs can also significantly enhance model explanatory power, although their strength may be influenced by specific contexts [49]. Based on this, this study selects perceived convenience, perceived quality, perceived risk, and perceived trust, not randomly combining variables, but because these four constructs correspond to the four core judgments made by elderly users when using smartphone mobile medical applications: whether it is time-saving and effortless, whether the platform and information are reliable, whether there are privacy and security risks in the usage process, and whether the platform and service providers are trustworthy [54,57]. More specifically, perceived risk and trust are the most common and necessary supplementary constructs in medical digital platforms. Chinese patient studies have shown that privacy risk and performance risk significantly reduce trust and adoption intention, while trust positively influences mHealth usage intention [60]. The reason why perceived quality is necessary is that, compared to general consumer apps, mobile medical applications essentially undertake functions of information transmission, service connection, and medical decision support; application quality and service quality affect users’ overall judgment of the platform's credibility and usability, and in the Chinese elderly population, service quality has been found to affect technological trust positively [54,83]. Perceived convenience is necessary because the medical treatment scenario itself involves high time costs, process complexity, and physical burden. For elderly users, whether appointment, payment, report query, and follow-up communication are time-saving and effortless is often a prerequisite for them to judge whether the platform is “useful” and whether it is worth adopting; relevant studies have shown that improving accessibility, process convenience, and saving time will enhance perceived usefulness and attitude [54,84,85]. Based on the above literature context, this study is not a simple repetition and verification of the existing TAM model. However, in response to the still relatively insufficient research scene of smartphone mobile medical application adoption by Chinese elderly people, beyond the core path of TAM, it introduces four constructs highly related to medical service platforms, namely convenience, quality, risk, and trust, to enhance the model's explanatory power for the usage intention of elderly users and more accurately respond to the real problem of the digital divide and family support in the Chinese context [49,54,86].

### Hypothesis development

#### Perceived ease of use.

In the TAM, perceived ease of use is defined as the degree to which an individual believes using a particular technology would be free from effort [34], and it positively affects perceived usefulness and attitude. A study [87] showed that doctors value the ease-of-use features of health apps the most. In the field of online health service systems, Lai et al. [88] and Kalayou et al. [89] confirmed that perceived ease of use could positively affect users’ attitudes. Meanwhile, Kalayou et al. [89] also confirmed a positive correlation between users’ perceived ease of use, perceived usefulness, and attitudes in their study of medical students’ use of mHealth apps. In addition, some studies [90,91] shows that users’ perception of the ease of use of technology increases users’ trust in technology. In the context of this study, perceived ease of use is understood as the degree to which using mHealth is considered effortless [9]. MHealth apps have been proven to be an easy-to-use intervention tool [92]. Therefore, this study uses this technology perception factor as a prerequisite for evaluating mobile health applications. Therefore, the following hypotheses are proposed in this study:

H1: Elderly people’s perceived ease of use of smartphone mHealth apps positively affects their perceived usefulness.

H2: Elderly people’s perceived ease of use of smartphone mHealth apps positively affects their attitudes.

H3: Elderly people’s perceived ease of use of smartphone mHealth apps positively affects their perceived trust.

#### Perceived usefulness.

Perceived usefulness is one of the important variables in the TAM. Perceived usefulness can be identified as the extent to which the user believes that using a new technology would improve his or her job performance or life [34]. Perceived usefulness is also one of the key factors affecting users’ technology adoption [93]. Wang, Fan [94] explored the factors that affect users’ continued use of mobile health applications and confirmed the impact of perceived usefulness. In technologies such as mobile phone mobile clinic systems [88] and electronic health systems [89], the impact of perceived usefulness on users’ attitudes has also been demonstrated. Jin and Yuan [9] revealed the utility of perceived usefulness in the use of mobile health applications. Moreover, the positive impact of perceived usefulness on users’ attitudes was also demonstrated in the study on health apps by Ma and Luo [2]. Wang and Zhang [95] discussed the function research of users’ use of the WeChat app for blood glucose management, revealing the usefulness of the WeChat app. Therefore, perceived usefulness for smartphone mHealth apps may also have an impact on attitudes towards their mHealth apps. Therefore, this study hypothesizes as follows:

H4: Elderly people’s perceived usefulness of smartphone mHealth apps positively affects their attitudes.

#### Attitude.

Attitude is a user’s negative or positive feeling towards a technology system or service, which can affect the user’s technology behavioral intention [35]. TAM [34] believed that individuals’ attitudes towards new technologies are influenced by their perceived usefulness and ease of use of the technologies, and attitudes further affect users’ intentions to use technologies. Ajzen [96] reported that users’ attitudes can directly affect their behavioral intentions. Gu et al. [97] and Guo et al. [98] confirmed the positive effect between patients’ attitudes and their continuance intention to use mHealth services. Liu et al. [99] revealed the positive impact of user attitudes on their intention to use mobile health applications. Uncovska and Freitag [10] discussed the acceptance of mobile health applications by German patients and the influencing factors of their intention to use them, and the results confirmed the importance of patient attitude. Therefore, this study believes that the elderly people’s attitudes towards smartphone mHealth apps will affect their intention to use these apps. In other words, the more positive the elderly users’ attitudes are, the higher their intentions to use smartphone mHealth app are likely to be. Thus, the following hypothesis is proposed:

H5: Elderly people’s attitudes towards smartphone mHealth apps positively affect their behavioral intentions.

#### Perceived risk.

Perceived risk was initially defined as consumers’ sense of uncertainty arising from the unpredictability of purchase outcomes [100]. In the context of technology adoption, it specifically refers to the extent to which potential users perceive a technology (such as a mobile banking application) as dangerous or likely to result in loss [82]. mHealth is based on internet technology, and users’ personal and private information, such as background information and access information, may be collected, tracked, and sold in the context of internet business [101]. Therefore, because mHealth services operate in a virtual network environment, they may involve risks [99]. For mHealth apps, users’ risk perception mainly focuses on privacy, psychological and financial risks [99]. Therefore, users are concerned about privacy. Without doubt, different users may focus on different risks. In the midst of the COVID-19 pandemic, a study of 1,003 German adults showed that they were very willing to share their health and medical data [102], demonstrating that attitudes towards personal data privacy and risk are also changing. Previous studies [103] explored the impact of risk on users’ technology adoption. Park et al. [104] pointed out that when users perceive the risks of information technology, they would have some uncertainties and worries and feel anxious about information technology. Kuhmeier and Knight [105] found that perceived risk has a significant negative impact on users’ purchase intention. Liu et al. [99] reported that the possible risks of mHealth negatively affected users’ attitudes towards and intentions to adopt it, and further affected users’ adoption behavior. Further, Li et al. [106] showed that perceived information risk negatively affected patients’ attitudes towards the use of online consultation services in online hospitals, and perceived medical risk network negatively affected the patients’ intentions to adopt online consultation services in online hospitals. Therefore, the following hypotheses are proposed in this study:

H6: Perceived risk has a significant negative impact on elderly people’s attitudes towards smartphone mHealth apps.

H7: Perceived risk has a significant negative impact on elderly people’s intention to adopt smartphone mHealth apps.

#### Perceived quality.

Users’ perception of technology or service quality needs concern. Some previous studies have emphasized perceived service quality [107] and online website quality [108] in relevant contexts. Tarkang et al. [109] pointed out that the quality of an app is a factor influencing users’ use or purchase of a technology or service online. Pee et al. [110] reported in their study that the information quality of a website or app technology product can improve users’ trust. Based on the stimulus-organism-response (SOR) theoretical framework, Dogra et al. [111] revealed the positive impact of app quality on users’ trust and online takeout purchase intention. Considering website design quality as an environmental cue, Nam et al. [112] revealed its positive impact on users’ trust and purchase intention. In this study, the quality of smartphone mHealth apps, a popular information service platform, is particularly important because it is an important connection medium between hospitals, doctors, and patients. Therefore, the following hypotheses are proposed in this study:

H8: Perceived quality positively affects elderly people’s trust in smartphone mHealth apps.

H9: Perceived quality positively affects elderly people’s intentions to use smartphone mHealth apps.

#### Perceived trust.

Trust can be regarded as one of the important emotional variables to maintain a lasting relationship between users and technology products [20]. The particularity of mHealth services makes users’ trust play a key role in their technology adoption behavior [113]. Doney and Cannon [114] pointed out that in the face of mHealth apps, users’ trust can reduce their uncertainty and demonstrate users’ acceptance of mHealth app services. Liu et al. [115] believed that a strong intermediary online platform can reduce the uncertainty in the process of online services, thereby increasing users’ intention to use online services. He [20] found that users’ trust in a mHealth platform would have a positive impact on users’ intentions to use the platform. Liu et al. [113] found that trust has a significant positive impact on users’ intentions to participate in and recommend mHealth apps. In their study on mHealth platforms, Octavius and Antonio [37] revealed that trust positively affected users’ behavioral intentions. Fitrianie et al. [116] suggested that mobile apps could be used as a potential protocol for cognitive behavioral therapy, and their study revealed that users’ trust was positively correlated with their behavioral intentions when using cognitive behavioral therapy apps. In addition, Alam et al. [117] confirmed in their study the significant impact of trust on users’ behavioral intention. When consumers have high trust in the platform, they are more willing to use mHealth apps. Due to the particularity of medical services and the personal information registration in the service process, users’ trust is very important in the research on user behavior of mHealth apps. Therefore, the following research hypothesis is proposed in this study:

H10: Elderly people’s perceived trust in smartphone mHealth apps has a positive impact on their intentions to use these apps.

#### Perceived convenience.

Okazaki and Mendez [118] interpreted perceived convenience as agility, accessibility, and flexibility of a service, which is flexible in time and location. When a product or service reduces the users’ physical, emotional, and cognitive burden, the product or service will be considered convenient [119]. To use traditional medical services, patients or their family members need to register, sign in, queue up, pay at the counter, etc., which requires a lot of time and energy. The inconvenience of these traditional medical services changes users’ attitudes and drives them to shift to mHealth [99]. Lai et al. [120] pointed out that the inconvenience in traditional offline medical services affected users’ attitudes toward online medical services. The more users recognize the convenience attribute of mHealth services, the more positive their attitudes toward mHealth services will be [99]. Chang et al. [119] and Li et al. [106] confirmed the positive impact of perceived convenience on users’ attitudes. Through mHealth apps, users can make online appointments and register, pay fees online, view inspection reports, and reduce their queuing time, which brings a convenient experience to them [121]. When users perceive that accessing medical services is convenient and takes less time and effort, they have a positive attitude toward participating in mHealth [122]. Yoon and Kim [123] found that perceived convenience had a positive impact on perceived usefulness. Also, in their study on the intention to use medical wearable technology, Zhang et al. [70] confirmed that perceived convenience had a positive impact on perceived usefulness. Based on the above, this study proposes the following hypotheses about perceived convenience:

H11: Perceived convenience of smartphone mHealth app has a positive impact on perceived usefulness.

H12: Perceived convenience of smartphone mHealth app has a positive impact on users’ attitudes.

In summary, this study proposes a model of elderly people’s adoption of smartphone mHealth apps, as shown in Fig 1. The conceptual model contains 12 research hypotheses.

**Fig 1:**
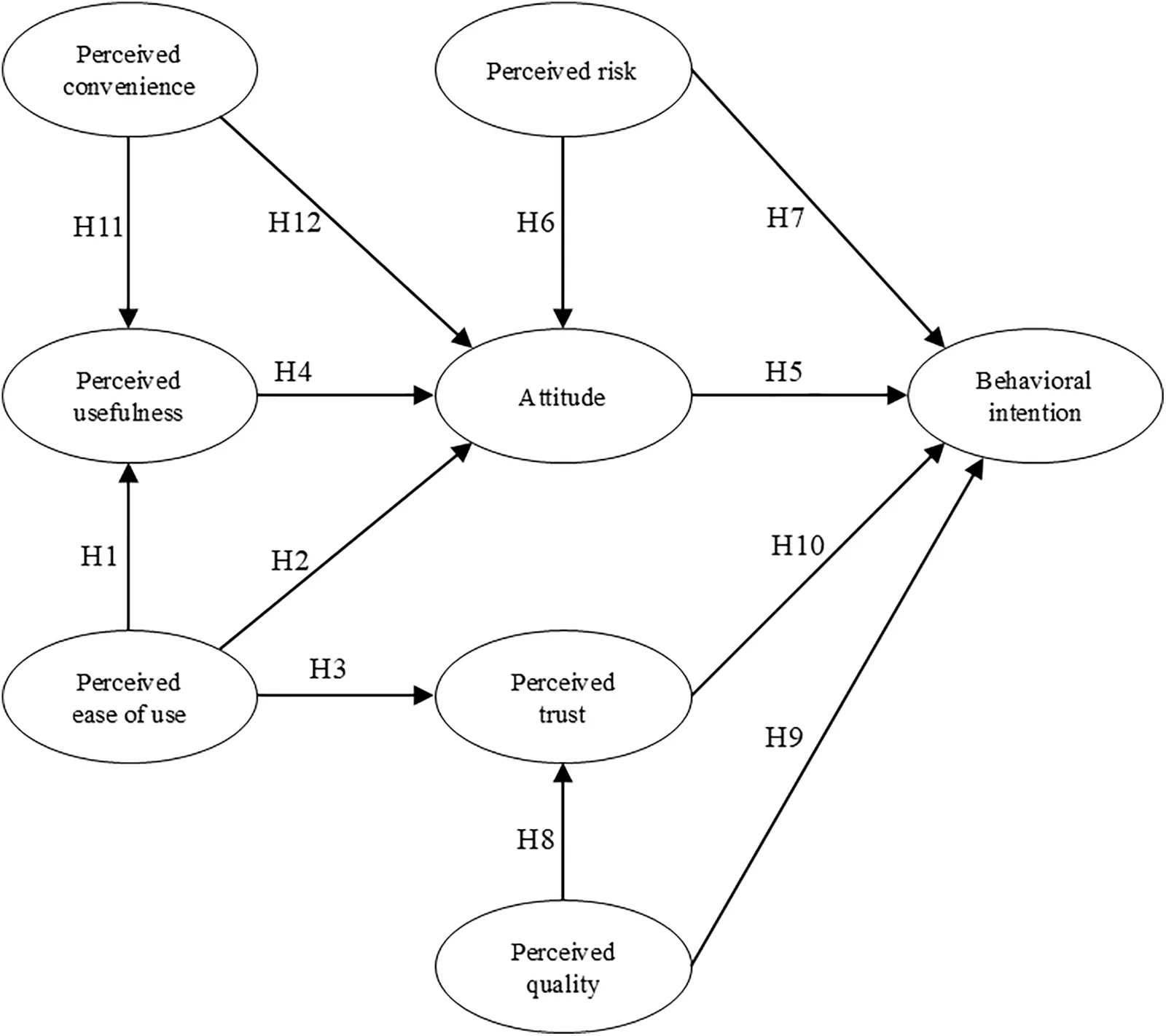
Model of Elderly People’s Adoption of Smartphone mHealth Apps.

## Methodology

### Research subjects and recruitment process

The recruitment criteria for this study were that the participants should be aged 60 or above and have experience in using smartphones and mobile medical applications. The researchers used purposive sampling to recruit respondents from residential areas and urban communities in Zhanjiang City, Guangdong Province. Zhanjiang City was chosen as the research location for demographic reasons: According to the seventh national population census bulletin of Guangdong Province, Guangdong is the province with the largest population in China. As of November 1, 2020, the proportion of the elderly population aged 60 and above among the province's permanent residents was 12.35%; among them, the proportion of the elderly population aged 60 and above in Zhanjiang City was 16.79%, ranking fifth among the 21 prefecture-level cities in the province, indicating a relatively prominent aging degree. Therefore, using the elderly population in Zhanjiang City as the research object can, to some extent, reflect the characteristics of the elderly population in regions with a higher aging degree in China, and the obtained samples have certain representativeness.

The ethical application for this research was reviewed and approved by the Academic Committee of Guangdong Ocean University on September 9, 2023. The approval covered the research purpose, design, methods, participant recruitment procedures, and data collection plan. During the actual implementation, after initially selecting the respondents, the researchers consulted their basic information such as age to determine if they met the recruitment criteria for this study. Before the formal questionnaire filling, the researchers first explained in detail the purpose, content, and participation method of this study to the respondents, and clearly informed them that their participation was completely voluntary, that the data would be strictly anonymized, that they could withdraw at any time without any consequences, and that they would not bear any responsibility. The research process did not involve any invasive operations or the collection of sensitive personal information. After the respondents fully understood the above content and verbally agreed, they could start the distribution and filling of the questionnaires. Since this research was an anonymous and low-risk questionnaire survey and did not collect any identification information of the respondents, to record the process of informed consent, the on-site investigators registered the completed informed consent forms and recorded only non-identifying information such as the questionnaire number. Considering that elderly respondents had concerns about taking photos or having their identities recorded, the research team did not conduct on-site photography and did not collect personal identification information such as names, ID numbers, and contact details to protect the privacy of the respondents and alleviate their concerns about participation. All participants were elderly individuals with independent living abilities, autonomous decision-making abilities, and independent judgment abilities, and could make their participation decisions independently after fully understanding the research content. The research questionnaire and data were processed anonymously, and all respondents only received oral thanks and wishes from the researchers and did not receive any form of material incentives or compensation.

From September 20th to October 25th, 2023, a total of 288 questionnaires were distributed, and 288 were retrieved, with a 100% recovery rate. The researchers screened the retrieved questionnaires based on the following criteria: eliminating those with a large number of missing or blank questions; eliminating those where all questions had the same option selected and showed obvious regularity in the answers. After the above screening, a total of 25 invalid questionnaires were eliminated, and 263 valid questionnaires were finally included for data analysis. A total of 263 valid questionnaires were recovered in this study, with 24 questions in each questionnaire. Kline [124] believes that the ratio of the number of measurement questions to the number of questionnaires should reach a standard of 5–10 times. Additionally, according to the tenfold rule proposed by Chin et al. [125], the minimum sample size required for PLS-SEM in the structural model is ten times the maximum path number pointing to any endogenous construct. In this study model, the maximum path number pointing to a single endogenous construct is 4, so the minimum sample size requirement is 40. In conclusion, the sample size of this study meets the standards.

### Research tools

A paper questionnaire was used to collect data. The questionnaire consisted of two parts, namely a scale and personal information. Specifically, the personal information included gender, age, educational level, smartphone use experience, and the times of mHealth app use in the past year. The scale was adapted from previous theoretical literature in the context of this study. The scale included 8 variables and 24 measurement questions, as shown in the Supporting information (2). More specifically, perceived usefulness and perceived ease of use were adapted from Davis [34], and behavioral intention was referred to Daghan and Akkoyunlu [126], with three questions in total; perceived convenience was referred to previous studies [123,127]; attitude was referred to the study by Taylor and Todd [128]; perceived quality included three questions, adapted from Agag and El-Masry [129]; the questions related to the perceived trust variable were also referred to some previous studies [130,131]; perceived risk was adapted from Deng et al. [132]. The questionnaire was measured using a 7-point Likert scale, with 1 indicating “totally disagree” and 7 indicating “totally agree”.

### Statistical approach

In this study, SPSS Statistics 25 software was used to sort out the research data, including missing value queries and descriptive statistical analysis. Structural Equation Model (SEM) is a widely accepted model for verifying theories with data [133]. Structural Equation models are mainly divided into covariance-based structural equation models (CB-SEM) and variance-based structural equation models (VB-SEM) [134]. Covariance-based structural equation model (CB-SEM) is a good method to analyze the relationship between predictive variables and potential variables. However, partial least squares (PLS) structural equation model (PLS-SEM) adopts a regression method to minimize the residual variation of endogenous variables [135]. Compared with CB-SEM, Leguina [136] pointed out that partial least squares structural equation modeling is fit for model development and can evaluate complex models [137]. Because partial least squares structural equation modeling does not require normality of research data [138], this study used partial least squares structural equation modeling to evaluate measurement models, structural models, and research hypotheses. The analysis tool used is SmartPLS 3. Among them, 5000 repeated samples were carried out using the bootstrapping method [139] to evaluate the path coefficients and their significance in the model.

## Results

### Demographics

This study collected 263 valid questionnaires. The questionnaire data are presented in the Supporting Information (S2 File). In terms of gender distribution, 136 male respondents (51.7%) and 127 female respondents (48.3%) were included. The gender ratio was relatively balanced. In terms of age distribution, respondents aged 60–69 were the most, with 147 people (55.9%); those aged 70–79 numbered 100 (38.0%); and those aged 80 and above numbered 16 (6.1%). The sample was mainly composed of younger elderly groups. In terms of educational attainment, 155 respondents had a junior high school or lower education level, accounting for 58.9%, which was the largest proportion group; 88 respondents had a high school education level, accounting for 33.5%; and 20 respondents had a bachelor's degree or higher, accounting for 7.6%. Overall, the educational attainment was relatively low. In terms of smartphone usage experience, the respondents with a usage period of 3–5 years were the most, with a total of 99 people, accounting for 37.6%; those with a usage period of more than 5 years had 103 people, accounting for 39.2%; those with a usage period of 1–3 years had 44 people, accounting for 16.7%; and those with a usage period of 0–1 year had the fewest, with only 17 people, accounting for 6.5%. This indicates that most respondents in the sample already had a certain foundation in smartphone usage. In terms of the frequency of using mobile medical applications in the past year, those who used it 4–6 times were the most, with a total of 106 people, accounting for 40.3%; those who used it 1–3 times had 95 people, accounting for 36.1%; those who used it 7–9 times had 48 people, accounting for 18.3%; and those who used it 10 times or more had 14 people, accounting for 5.3%. This indicates that respondents’ overall usage frequency of mobile medical applications was at a medium-low level.

### Common method bias

Common method bias is a focus of this study and can be reduced and evaluated in several ways. First, according to the suggestions made by Hew et al. [140], the researchers prepared anonymous questionnaire questions in concise language to minimize common method bias. Specifically, respondents were explicitly informed of the anonymous and confidential nature of the survey in the questionnaire preamble, and the researchers verbally reiterated these assurances before distributing the questionnaires to alleviate respondents’ concerns and encourage truthful responses. Furthermore, the wording of all questionnaire items was carefully crafted to be concise and unambiguous, taking into account the comprehension level and contextual familiarity of elderly respondents, thereby minimizing systematic response bias arising from item ambiguity.

Next, according to the suggestions by Podsakoff et al. [141], the researchers conducted Harman single-factor analysis on the questionnaire data. The results showed that the largest single factor explained only 41.898% of the total variance, which did not exceed the standard of 50%, indicating that there was no problem of common method bias in this study. Furthermore, we employed the full collinearity assessment approach based on the Variance Inflation Factor (VIF) as proposed by Kock [142]. Kock [142] suggested that when the VIF values of all constructs are less than or equal to 3.3, the model can be considered free from significant common method bias. As presented in Table 1, the VIF values of all constructs in this study ranged from 1.307 to 2.207, all of which fall substantially below the threshold of 3.3, providing further evidence that the influence of common method bias on the findings of this study is negligible. Taken together, the combination of procedural controls and multiple statistical tests confirms that common method bias does not pose a significant threat to this study's conclusions.

**Table 1:** Variance inflation factor.

	Attitude	Behavioral intention	Perceived trust	Perceived usefulness
Attitude		1.314		
Perceived convenience	2.207			1.556
Perceived ease of use	1.717		1.307	1.556
Perceived quality		1.576	1.307	
Perceived risk	1.354	1.379		
Perceived trust		1.703		
Perceived usefulness	1.540			

### Measurement model

Regarding reliability test, Anderson and Gerbing [143] believed that Cronbach’s alpha should be greater than 0.7. Table 2 shows that the minimum value of Cronbach’s alpha was 0.751, indicating that the reliability was up to the standard. According to Chin [144], the combination reliability should be greater than 0.7 to meet the reliability requirements. Table 2 shows that the composite reliability was above 0.8, indicating a high composite reliability. Nunnally [145] pointed out that when the average variance extracted (AVE) exceeded 0.5, the threshold for the convergence validity of the research model was reached. Table 2 shows that the average variance extracted (AVE) of all variables in this study ranged from 0.667 to 0.841, all of which were greater than 0.5, reaching the standard of convergence validity. Meanwhile, the Heterotrait-Monotrait Ratio (HTMT), as an evaluation index of discriminant validity, needs to meet the threshold that HTMT is less than 0.9 [146]. Table 3 shows that all HTMT values do not exceed 0.9, indicating that the model constructs have differential validity. Furthermore, Fornell and Larcker [147] pointed out that when the square root of the AVE of each variable is higher than its correlation coefficient, it indicates that the questionnaire has discriminant validity. As shown by the diagonal AVE values in Table 4, the variables in this study meet the discriminant validity standard of the Fornell-Larcker criterion. In summary, the reliability and validity of the measurement model in this study meet the requirements.

**Table 2:** Construct Reliability and Validity.

	Cronbach's Alpha	Composite Reliability	Average Variance Extracted
Attitude	0.751	0.857	0.667
Behavioral intention	0.858	0.913	0.778
Perceived convenience	0.899	0.937	0.832
Perceived ease of use	0.905	0.941	0.841
Perceived quality	0.845	0.906	0.763
Perceived risk	0.833	0.897	0.744
Perceived trust	0.796	0.873	0.696
Perceived usefulness	0.823	0.895	0.739

**Table 3:** Discriminant validity based on Heterotrait-Monotrait Ratio.

	Attitude	Behavioral intention	Perceived convenience	Perceived ease of use	Perceived quality	Perceived risk	Perceived trust	Perceived usefulness
Attitude								
Behavioral intention	0.660							
Perceived convenience	0.610	0.719						
Perceived ease of use	0.471	0.637	0.663					
Perceived quality	0.561	0.664	0.658	0.548				
Perceived risk	0.310	0.241	0.556	0.245	0.446			
Perceived trust	0.488	0.634	0.727	0.448	0.629	0.643		
Perceived usefulness	0.630	0.777	0.627	0.600	0.692	0.271	0.548	

**Table 4:** Discriminant Validity—Fornell-Larcker Criterion.

	Attitude	Behavioral intention	Perceived convenience	Perceived ease of use	Perceived quality	Perceived risk	Perceived trust	Perceived usefulness
Attitude	**0.816**							
Behavioral intention	0.537	**0.882**						
Perceived convenience	0.509	0.630	**0.912**					
Perceived ease of use	0.400	0.564	0.598	**0.917**				
Perceived quality	0.452	0.568	0.575	0.485	**0.873**			
Perceived risk	0.253	0.222	0.502	0.221	0.383	**0.863**		
Perceived trust	0.398	0.601	0.622	0.414	0.533	0.507	**0.834**	
Perceived usefulness	0.508	0.653	0.540	0.518	0.578	0.241	0.479	**0.860**

Note. a. The bold number is the square root of AVE.

### Structural model

The coefficient of determination (R2) is an important index for evaluating the explanatory power of a research model. Falk and Miller [148] suggested that when the coefficient of determination R^2^ is greater than 10%, it indicates independent explanatory power. Table 5 shows that each factor variable has explanatory power, and the R2 value for behavioral intention is as high as 52.9%.

**Table 5:** Coefficients of determination.

	R Square
Attitude	0.338
Behavioral intention	0.529
Perceived trust	0.316
Perceived usefulness	0.351

In terms of model fit, current guidance from Hair et al. [146] and Hu and Bentler [149] recommends the standardized root mean square error of approximation (SRMR) as the primary indicator for evaluating overall model fit in PLS-SEM. The SRMR value in this study is 0.071, which is below the critical standard of 0.08, indicating that the model fits well overall. Additionally, for the goodness-of-fit (GOF) indicators, Tenenhaus [150] considers 0.36 the standard for a model with high fit. The GOF value in this study is 0.538, which is higher than this critical value and also corroborates the above conclusion. Therefore, the model has a good fit.

Meanwhile, according to [146], the Q2 value can be regarded as an important indicator for evaluating the model's predictive correlation. Specifically, Chin [151] divided the Q2 value into three levels: when the Q2 value exceeds 0.35, it indicates that the model has a strong predictive correlation; between 0.15 and 0.35, it represents moderate predictive correlation; and above 0.02 but below 0.15, it reflects a relatively weak predictive correlation. In this study, as shown in Table 6, the Q2 value for the elderly's intention to use smartphone mobile medical applications reached 0.395, exceeding the critical value of 0.35. Thus, the model in this study has a relatively high level of predictive correlation.

**Table 6:** Prediction Correlation.

	Q² (=1-SSE/SSO)
Attitude	0.216
Behavioral intention	0.395
Perceived trust	0.203
Perceived usefulness	0.254

Additionally, the effect value f2 is used to measure the degree of influence of extrinsic constructs on the intrinsic construct [152]. This further evaluates the explanatory power of each extrinsic construct on the intrinsic construct. According to the judgment criteria proposed by Cohen [152], when the f2 value is approximately 0.02, 0.15, and 0.35, it corresponds to weak, medium, and strong explanatory capabilities, respectively. From the results in Table 7, the f² value of Attitude on Behavioral Intention is 0.131, which belongs to moderate explanatory power; the f² values of Perceived Convenience on Perceived Usefulness and Attitude are 0.127 and 0.061 respectively, which are between weak and medium explanatory power; the f² values of Perceived Ease of Use on Perceived Usefulness, Attitude, and Perceived Trust successively are 0.091, 0.003, and 0.047, which overall leans towards weak explanatory power; the f² values of Perceived Quality on Perceived Trust and Behavioral Intention are 0.211 and 0.104 respectively, the former has reached medium to strong explanatory power, and the latter is at the weak to medium level; the f² value of Perceived Risk on Attitude and Behavioral Intention is 0.000 and 0.046 respectively, which has almost no explanatory power to weak explanatory power; the f² value of Perceived Trust on Behavioral Intention is 0.228, which has reached medium explanatory power; the f² value of Perceived Usefulness on Attitude is 0.097, which belongs to weak to medium explanatory power. In summary, the f² values between the various constructs are generally between weak and medium explanatory power. Among them, Perceived Trust and Perceived Quality have relatively prominent explanatory effects on Behavioral Intention and Perceived Trust, indicating that the various constructs in the research model have a certain degree of explanatory effect, and the model as a whole has considerable rationality and explanatory value.

**Table 7:** Effect Size f².

	Attitude	Behavioral intention	Perceived trust	Perceived usefulness
Attitude		0.131		
Perceived convenience	0.061			0.127
Perceived ease of use	0.003		0.047	0.091
Perceived quality		0.104	0.211	
Perceived risk	0.000	0.046		
Perceived trust		0.228		
Perceived usefulness	0.097			

This study conducted repeated sampling 5,000 times using the bootstrapping method [139] to evaluate the model path coefficients and their significance. The results are shown in Fig 2 and Table 8. As shown, perceived ease of use positively affects perceived usefulness (β = 0.304, P < .001) and perceived trust (β = 0.204, P < 0.001); therefore, H1 and H3 are supported. Perceived usefulness positively affects attitude (β = 0.315, *P* < 0.001), so H4 is supported. Perceived quality has a direct positive impact on perceived trust (β = 0.434, *P* < 0.001) and behavioral intention (β = 0.278, *P* < 0.001), so H8 and H9 are valid. Perceived convenience also positively affects perceived usefulness (β = 0.358, *P* < 0.001) and attitude (β = 0.299, *P* < 0.001), so H11 and H12 are valid. In addition, perceived trust (β = 0.427, *P* < 0.001), perceived risk (β = −0.173, *P* < 0.001), and attitude (β = 0.285, *P* < 0.001) all have a direct impact on older adults’ intentions to use smartphone mHealth apps, so H10, H7, and H5 are supported. However, perceived ease of use (β = 0.055, *P* = 0.359) and perceived risk (β = 0.015, *P* = 0.839) do not affect users’ attitude, so H2 and H6 are not supported.

**Table 8:** Hypothesis test results.

Hypotheses	Original Sample	T Statistics	*P* Values	Result
H1: Perceived ease of use - > Perceived usefulness	0.304	4.416	0.000	Supported
H2: Perceived ease of use - > Attitude	0.055	0.917	0.359	Unsupported
H3: Perceived ease of use - > Perceived trust	0.204	3.567	0.000	Supported
H4: Perceived usefulness - > Attitude	0.315	4.859	0.000	Supported
H5: Attitude - > Behavioral intention	0.285	5.199	0.000	Supported
H6: Perceived risk - > Attitude	0.015	0.204	0.839	Unsupported
H7: Perceived risk - > Behavioral intention	−0.173	3.316	0.001	Supported
H8: Perceived quality - > Perceived trust	0.434	7.021	0.000	Supported
H9: Perceived quality - > Behavioral intention	0.278	4.997	0.000	Supported
H10: Perceived trust - > Behavioral intention	0.427	5.858	0.000	Supported
H11: Perceived convenience - > Perceived usefulness	0.358	4.856	0.000	Supported
H12: Perceived convenience - > Attitude	0.299	3.782	0.000	Supported

**Fig 2:**
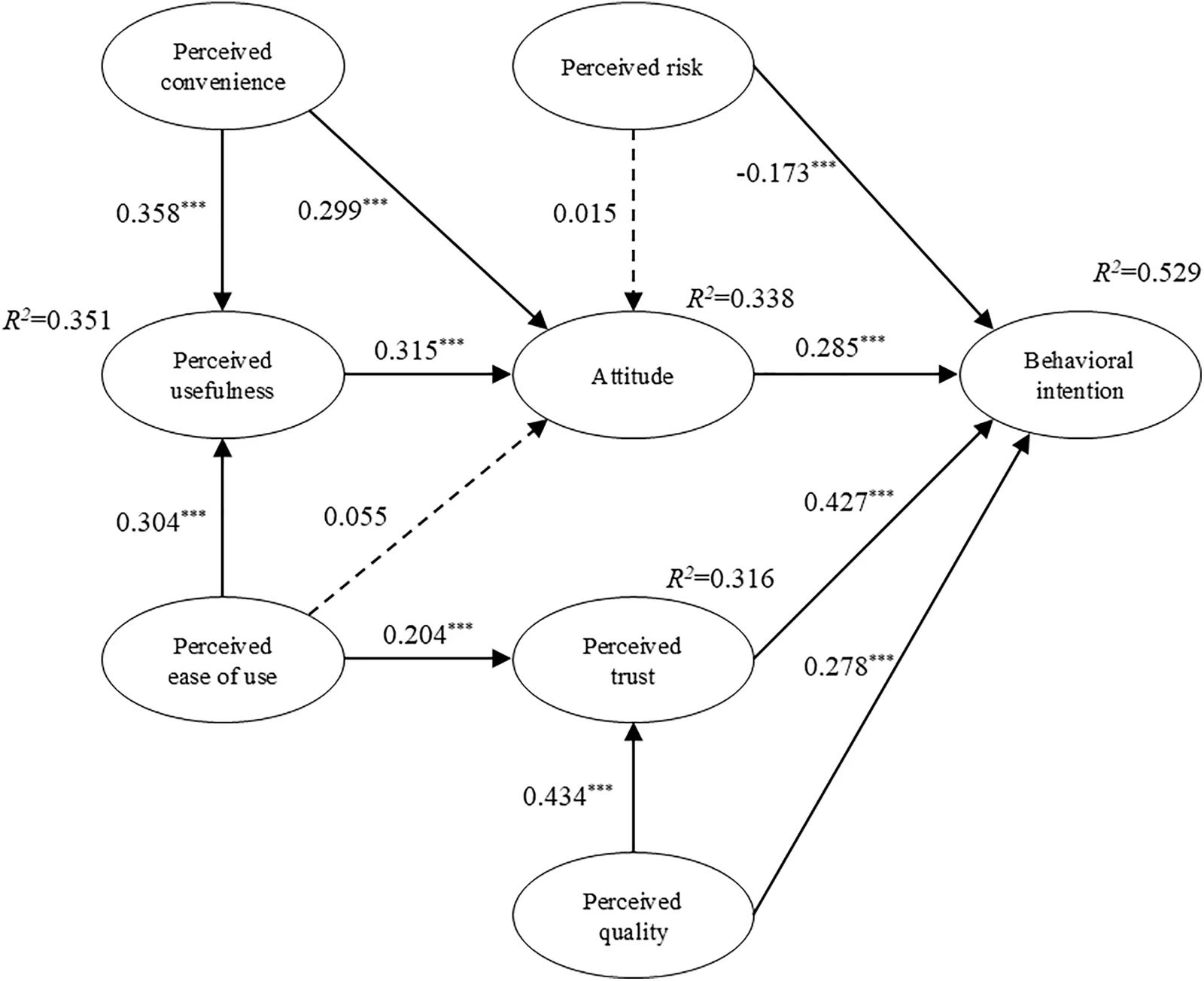
Evaluation Results of the Model of Elderly People’s Adoption of Smartphone mHealth Apps.

## Discussion and contribution

### Discussion

The healthcare system is shifting toward mobile healthcare (mHealth) platforms accessible to a wide range of users. Mobile healthcare can support both health management and efficient medical services, making it an important digital medium for connecting patients with medical institutions. Existing studies show that the key mechanism for the elderly's adoption of health technologies is unstable and varies with the type of technology, regional culture, and usage experience. Therefore, the analysis of elderly people's intention to adopt smartphone mobile healthcare applications needs to be explained in a specific context [61,153]. This study, based on the technology acceptance model, examines the relationships between perceived convenience, perceived quality, perceived risk, perceived trust, perceived usefulness, perceived ease of use, and attitude. The results show that the use intention of older adults for smartphone mobile healthcare applications is mainly influenced by perceived trust, attitude, perceived quality, and perceived risk, with perceived trust having the strongest effect; at the same time, perceived quality and perceived ease of use directly enhance perceived trust, while perceived convenience positively affects perceived usefulness and attitude. This means that beyond statistical significance, this study also reveals a practical priority hierarchy: for the elderly, whether it is “worthy of trust” directly determines whether they are willing to adopt. At the same time, convenience more indirectly affects usefulness perception and attitude through enhancing usability perception and positive attitude [153–155].

This study first found that perceived trust significantly and positively influences the use intention of the elderly, consistent with existing studies on elderly mHealth. A study of Hong Kong elderly people showed that trust is an important missing variable in explaining mHealth adoption intention, and service quality, social influence, and government policies jointly shape technological trust [79]. A study of elderly people in the Netherlands also showed that trust has the largest correlation with mHealth use intention and can explain over 20% of the variance in intention [154]. This indicates that in healthcare service platforms, elderly users are not just evaluating a “tool,” but rather evaluating the doctors, institutions, data processing mechanisms, and platform responsibility boundaries behind the technology. Therefore, trust is closer to the adoption premise than the technical preference in general consumer applications [154]. From a theoretical perspective, this result also supports the approach of regarding trust as a core belief layer concept in the context of elderly mHealth, rather than an edge supplementary variable, because perceived usefulness often builds on trust in the accuracy and reliability of functions [62].

Secondly, perceived risk significantly negatively influences the use intention, which is consistent with existing studies. A study of elderly people in the Netherlands showed that privacy risks, performance risks, and legal concerns all significantly reduce mHealth use intention, while trust has the opposite effect [154]. Similar findings have been reported in studies of the elderly population in China and other studies on elderly technology adoption, showing that perceived risk inhibits behavioral intention and often works by weakening trust or increasing uncertainty [156,157]. However, the path “perceived risk → attitude” in this study was not significant. This result cannot be simply attributed to the “peculiarities of the elderly,” but is more likely to reflect the experience screening effect: elderly users with prior use experience often cross the initial risk threshold before forming a general attitude, so risk may not continue to directly shape their attitude, but still has an inhibitory effect on whether they are willing to continue or ultimately adopt. A study in Thailand showed that elderly people with prior mHealth experience are more likely to consider the benefits to be greater than the risks and challenges, but if they have greater concerns about data security, privacy, and effectiveness, their use intention will still decrease [158]. This also explains why risk has a significant effect on intention but not on attitude: risk in this study sample is more like a final decision constraint rather than a stable emotional evaluation source.

Again, perceived ease of use did not significantly affect attitude. This result differs from some studies, but it is not without a literature basis. However, some studies suggest that in the context of elderly health technology, the effect of perceived ease of use often manifests indirectly through usefulness or value rather than directly on attitude. The latest meta-analysis indicates that one of the important roles of perceived usability in adoption is to achieve this through enhancing perceived usefulness [153]; studies on elderly mHealth applications have also found that perceived ease of use significantly enhances perceived usefulness, which in turn affects intention [159,160]. Considering the characteristics of the elderly population, this non-significant result may also be related to proxy operations and supportive use. Existing research has pointed out that elderly people often rely on family support, training, or community guidance to overcome initial operational obstacles; in such cases, their judgment of “whether it is easy to operate” may not directly translate into personal attitude, but is first absorbed by external support and ultimately manifested as an increase in usefulness, confidence, or trust [62,79,161]. In other words, when children, medical staff, or community volunteers provide alternative assistance in registration, payment, report queries, and other aspects, elderly users may not form a more positive attitude because “they can operate it,” but will recognize the usefulness and credibility of the technology because “it can be accomplished.”

This study also found that perceived quality significantly enhances perceived trust, which reinforces the attribute of mobile healthcare as a “service platform” rather than a simple “technical tool.” Elderly mHealth studies have shown that high-quality information, reliable functions, and good service performance can enhance the perception of usefulness and adoption intention [160]. The Hong Kong study further pointed out that service quality is one of the key antecedents for forming technical trust [79,154]. This means that the elderly do not narrowly understand “quality” as interface aesthetics, but rather value whether the diagnosis and treatment information is accurate, the process is stable, the feedback is timely, and the medical entity behind it is reliable. This result also explains why, in high-sensitivity health information scenarios, quality does not remain at the satisfaction level but further transforms into a trust judgment [62].

Furthermore, perceived convenience positively affects perceived usefulness and attitude. Here, it is necessary to clarify the boundaries between convenience and usefulness further. In this study, perceived convenience emphasizes time-saving, effort-saving, less running around, and the ability to complete tasks anytime and anywhere, which belongs to an external perception closer to the attributes of the service process. In contrast, perceived usefulness emphasizes whether the application can improve health management efficiency, improve medical outcomes, and bring substantive benefits, which belongs to a general judgment of the value of the outcome [58,162]. The two are related but not equivalent: convenience answers “is it easier to handle,” while usefulness answers “is this worth using.” This causal distinction is also supported by existing research; that is, elderly users usually perceive convenience from accessibility, process simplification, and time-saving, and then infer whether the technology is useful [62,162]. Therefore, in this study, convenience affects usefulness rather than being measured alongside usefulness, and it has clear theoretical boundaries.

From a theoretical perspective, the results of this study do not support the simple understanding of elderly mHealth adoption as a linear replication of the traditional TAM path. The meta-analysis shows significant heterogeneity in the adoption of health technologies among the elderly, and the relative importance of driving factors varies across technology categories. Digital health platforms rely more on perceived usefulness, while other assistive devices may rely more on usability or safety perceptions [61,153]. In this study, the prominent roles of trust, risk, and quality indicate that in the context of elderly smartphone mobile medical applications, service credibility and risk control are closer to the core of the adoption mechanism than mere operational simplification. This finding echoes some extended UTAUT studies: in some elderly mHealth research, effort expectancy or facilitating conditions are not always significant, while factors such as trust, service quality, and habits, which are more relevant to the actual use of services, are actually more crucial [79,163]. From a practical perspective, merely reporting “significant” is not enough; it is more important to prioritize interventions based on the magnitude of path coefficients. In this study, perceived trust has the greatest impact on the intention to use (β = 0.427), so the priority should be trust governance, including weakening unnecessary mandatory authorization for sensitive information, enhancing transparency of privacy notification, introducing third-party security certification, clarifying the responsibility boundaries between doctors and platforms, and providing understandable data usage feedback. This aligns with the elderly's high sensitivity to privacy, effectiveness, and doctor credibility [154,158]. The second priority is to enhance attitude (β = 0.285) and perceived quality (β = 0.278), with the focus not on increasing entertainment, but on improving functional reliability, information accuracy, timely reporting feedback, and task completion success rate, as the elderly view mHealth more as a functional health tool rather than an entertainment product [58,160]. The third priority is to reduce perceived risk (β = −0.173), and specific safeguard measures should be designed for privacy, performance, and financial security, as these risks directly lower the final adoption intention [154,156]. Finally, although convenience and ease of use mainly exert their effects through indirect paths, they should still be enhanced through simplifying registration, reducing steps, providing large-font interfaces, visual guidance, and family/community assistance to improve the perceived usefulness, trust, and positive attitudes, especially for low SES or low-experience elderly individuals [61,164].

Overall, this study demonstrates that the adoption of smartphone mobile medical applications by the elderly is not determined by a single technological factor, but is driven by a contextual mechanism composed of trust, risk, quality, and convenience. Compared to simply emphasizing interface ease of use, this study supports placing the promotion of elderly mHealth on the focus of building trustworthy services, controlling risk visibility, delivering key functions reliably, and designing convenience throughout the process. This aligns more closely with elderly users’ adoption logic and the essential attributes of digital health service platforms [153,164].

### Theoretical and practical implications

The theoretical contributions of this study are mainly reflected in three aspects. First, this research does not merely repeat the verification of the Technology Acceptance Model (TAM). Instead, based on the fact that the core path of the TAM has been stable in the research on elderly digital health adoption, and the significant differences in contexts have been revealed, it further responds to the problem of the insufficient explanatory power of the existing model in specific populations and specific technical scenarios; the existing integrated evidence indicates that perceived usefulness, perceived ease of use, social influence, although they generally affect the intention of elderly people to adopt health technologies, there is strong heterogeneity among different regions, technical types, and usage contexts. Therefore, the relevant conclusions need to be re-examined in specific contexts [61]. Second, while retaining the core logic of the TAM, this study introduces constructs such as perceived convenience, perceived quality, perceived risk, and perceived trust, and advances the traditional analysis framework that is biased towards general information technology acceptance to a smartphone mobile medical application scenario that combines technical attributes, service attributes, and health information sensitivity; this expansion direction is consistent with the conclusion of existing research that the TAM needs to enhance its explanatory power through contextual variables, especially factors such as trust, risk, service quality, interface characteristics, technical anxiety, and social support, which have been repeatedly proven to be important supplementary variables in the adoption of elderly health technologies [63,81]. Third, this study focuses on Chinese elderly smartphone users and pays attention to their intention to adopt mobile medical applications, thereby supplementing the insufficient attention of existing research to the Chinese context, elderly end-users, and the psychological mechanisms in the pre-adoption stage; existing research has pointed out that the adoption mechanism of elderly health technologies has obvious cultural and regional differences, and the pathways in China and other non-Western contexts cannot be directly extrapolated from Western research, and family dependence, social influence, service experience, and local medical environment will significantly shape the adoption logic of the elderly. Therefore, it is necessary to conduct more targeted theoretical tests and model expansions in the Chinese context [54,165,166]. In summary, this study constructs an extended model that is more in line with the usage context of Chinese elderly smartphone mobile medical applications, not only enriching the contextual application of the TAM in the field of elderly digital health, but also providing a theoretical basis for subsequent research to further distinguish the effects of different health technologies, different adoption stages, and different cultural backgrounds.

This study provides some practical implications that may facilitate the adoption of mHealth apps. First, this study provides empirical evidence for the age-friendly design of mobile health applications. The findings reveal that perceived ease of use, perceived convenience, and perceived quality are key factors driving adoption among elderly users, indicating that age-friendly design is not an optional supplementary feature but a core element influencing elderly users’ adoption decisions, thereby offering valuable directional guidance for app developers’ product design. For example, developers can meaningfully reduce the operational difficulty and cognitive burden for elderly users by supporting dynamic font size adjustment, simplifying page navigation hierarchies, and introducing slow-paced voice guidance, thus enhancing their perceived ease of use and convenience. Second, this study establishes the central role of trust mechanisms in the adoption process among elderly users. Perceived trust exerts the most significant influence on behavioral intention and is positively driven by perceived quality. The revelation of this pathway mechanism offers healthcare providers and developers a new perspective for understanding elderly users’ decision-making psychology, facilitating the formulation of more targeted service strategies in terms of professionalism and transparency. For example, healthcare institutions can display verified practitioner credentials on the platform, explicitly state data privacy protection policies, and have professional medical teams review health information content, treating the enhancement of perceived quality as an effective entry point for building user trust. Third, this study provides a reference framework for policymakers in advancing digital healthcare services for the aging population. The study identifies perceived risk as a critical barrier inhibiting elderly users’ adoption intention, suggesting that relevant institutions should prioritize institutional safeguards and digital health literacy cultivation when formulating digital healthcare promotion policies, thereby providing empirical support for setting policy-level priorities. For instance, hands-on smartphone medical application training programs can be delivered through community health service stations and elderly activity centers, alongside the establishment of data security certification standards for mobile health platforms, collaboratively reducing elderly users’ perceived risk from both skill development and institutional regulation perspectives. Fourth, the integrated model framework proposed in this study holds considerable practical reference value. By incorporating multiple dimensions of users’ psychological perceptions, the framework enables healthcare technology enterprises, hospital management institutions, and marketing professionals to understand elderly users’ behavioral logic from different perspectives, facilitating more user-centered decisions in product development, service management, and promotional practices. For example, marketing professionals can leverage authentic user experiences to drive word-of-mouth promotion in community or hospital waiting environments, while simultaneously targeting the adult children of elderly users through directed outreach, harnessing family trust relationships to generate an “intergenerational communication” effect, and positioning the construction of a professionally credible brand image as the core strategy for reaching elderly audiences, ultimately fostering the elderly population's active integration into the digital healthcare era.

### Limitations and future directions

This study has the following limitations that need to be further improved in future research. First, regarding the research subjects and generalizability, this study focused on elderly users in China and did not include family members or caregivers who may use mobile medical applications on behalf of the elderly; the conclusions are also based on the Chinese cultural context. The cross-cultural applicability needs to be verified. Future research can expand the scope of research subjects and conduct cross-cultural or cross-regional comparative studies. Secondly, in terms of sample representativeness, the sample in this study was obtained through purposive sampling from urban communities in Zhanjiang City. The respondents all had practical usage experience and had a low acceptance of digital technology or lived in rural areas, which led to insufficient representativeness of the elderly group with low usage of mobile medical applications and a limited sample size that may impose certain restrictions on statistical test power. Future research can adopt stratified sampling or random sampling methods to expand the geographical coverage and increase the sample size. Additionally, the sample in this study all came from elderly people with smartphones and experience in using mobile medical applications, so the research conclusions mainly apply to explaining the usage intentions of existing users, but cannot be directly extrapolated to elderly non-users who have never used such applications. Due to the fact that existing users have experienced a certain degree of technological contact and actual use, the sample may have selection bias; that is, it is more likely to retain individuals with higher digital skills, more positive attitudes, or those who have overcome initial usage barriers. Therefore, future research can include elderly people who have never used mobile medical applications to compare the differences in the formation mechanisms of initial usage intentions between non-users and existing users. Thirdly, in terms of research design and data sources, this study adopted a cross-sectional design and self-reported questionnaire data, which cannot infer causal relationships and dynamic changes between variables, and may also have potential problems such as social expectation bias. Future research can introduce a longitudinal tracking design and supplement with objective behavioral data. Fourthly, in terms of research variables, the mobile medical usage behavior of the elderly is influenced by multiple factors. Future research can further include variables such as digital literacy, health literacy, social influence, and facilitating conditions to construct a more complete explanatory framework.

## Conclusion

In this study, a model for elderly people’s acceptance of smartphone mHealth apps was constructed, which can be used to predict and explain users’ behavior in using mHealth apps. The results show that perceived convenience, perceived risk, perceived trust, perceived quality, perceived usefulness, perceived ease of use, and attitude are all factors driving users to adopt smartphone mHealth apps; perceived risk has a negative impact on intention to use mHealth apps. This work enriches the research on mHealth adoption, expands the technology acceptance model, and can provide references for improving users’ intentions to adopt mHealth apps.

## Supporting information

S1 File Measurement items. (DOCX)

S2 File Questionnaire data. (CSV)
