# Supplementary material for: Factors influencing elderly people’s intentions to use smartphone mHealth apps: Quantitative research
Source: PLoS One. 2026 Oct 23;21(10):e0361137. doi: 10.1371/journal.pone.0361137 (PMC13693932; doi:10.1371/journal.pone.0361137)
Supplement: Supplementary file 1 [file pone.0361137.s001.docx]

Multimedia Appendix 1: Measurement items

| Constructs | Items | Source |
| --- | --- | --- |
| Perceived convenience | I can use my smartphone mobile medical application anywhere.  I can use my smartphone to move medical applications at any time.  Smart phone mobile medical application can provide convenience for medical information query. | Gefen et al. [85]  Yoon et al. [82]; |
| Perceived quality | I think the smartphone mobile medical application provides enough information.  I think the smart phone mobile medical application provides reliable information.  I think the smartphone mobile medical application provides a reliable service. | El-Masry [87] |
| Perceived usefulness | Using a smartphone mobile medical application can meet my medical needs.  Using a smartphone mobile medical application can improve my medical efficiency.  Overall, the smartphone mobile medical application is very useful for my medical treatment. | Davis [19] |
| Perceived ease of use | I find it easy for me to operate mobile medical applications on smartphones.  It won't take me much time to operate the mobile medical application of smartphone.  Overall, I find the smartphone mobile medical application easy to use. | Davis [19] |
| Attitude | Using a smartphone mobile medical app is a good idea.  Using a smartphone mobile medical application is a wise idea.  I like the idea of using mobile medical apps on my smartphone. | Taylor & Todd [86] |
| Behavioral intention | I intend to use smartphone mobile medical applications in the future.  I will use smartphone mobile medical applications in the future.  I will regularly use smartphone mobile medical applications in the future. | Daghan & Akkoyunlu [84] |
| Perceived trust | I believe that the smartphone mobile medical application is trustworthy.  I believe that smartphone mobile medical applications are professional.  I believe that smartphone mobile medical applications are reliable. | McKnight et al [89];  Pavlou & Gefen [88] |
| Perceived risk | I think the smartphone mobile medical application may reveal my personal privacy.  I think the smartphone mobile medical application may use my personal information for other purposes.  I think there are risks in medical payment in smart phone mobile medical application. | Deng et al. [90] |
